# Knock-Down of Specific Thyroid Hormone Receptor Isoforms Impairs Body Plan Development in Zebrafish

**DOI:** 10.3389/fendo.2019.00156

**Published:** 2019-03-14

**Authors:** Iván Lazcano, Roberto Rodríguez-Ortiz, Patricia Villalobos, Ataúlfo Martínez-Torres, Juan Carlos Solís-Saínz, Aurea Orozco

**Affiliations:** ^1^Departamento de Neurobiología Celular y Molecular, Instituto de Neurobiología, Universidad Nacional Autónoma de México (UNAM), Querétaro, Mexico; ^2^Departmento de Investigación Biomédica, Facultad de Medicina, Universidad Autónoma de Querétaro, Querétaro, Mexico; ^3^CONACYT – Instituto de Neurobiología, Universidad Nacional Autónoma de México, Querétaro, Mexico

**Keywords:** thyroid hormone receptors, thyroid hormones, CRISPR/Cas9, development, zebrafish

## Abstract

The role of thyroid hormones (THs) in development has been extensively studied, however, the specific molecular mechanisms involved are far from being clear. THs act by binding to TH nuclear receptors (TR) that act as ligand-dependent transcription factors to regulate TH-dependent gene expression. Like vertebrates, zebrafish express different isoforms of functional Tr alpha and beta, some of which can bind alternative ligands like 3,5-T2. In this study, we first analyzed the effects of exogenous T3 and 3,5-T2 exposure during embryogenesis. The percentage of affected embryos was similar to those vehicle-injected, suggesting that the early exposure to low TH levels is not sufficient to elicit effects upon the phenotype of the embryo. We then generated crispants for four isoforms of *thr* to learn more about the role of these receptors in early development. We found that crispant larvae from *thraa* and a newly identified *l-thrb*+, but not *thrab* and canonical *thrb1* showed profound deleterious effects upon symmetry and laterality, suggesting early novel roles for these Tr isoforms in the body plan developmental program. Since critical events that determine cell fate start in the late gastrula, we tested if some genes that are expressed during early developmental stages could indeed be TH targets. We identify early development genes, like *sox10* and *eve*, that were specifically over-expressed in *thraa* and *l-thrb*+ crispants, suggesting that these specific *thr* isoforms function as transcription repressors for these genes, while transcription of *zic* and *ets* appear to be *thraa* and *l-thrb*+-mediated, respectively. Overall, present results show that TH signaling participates in early zebrafish development and identify Tr isoform-specific mediated regulation of early gene expression.

## Introduction

Thyroid hormones (THs) play important roles in different developmental processes and life transitional events of vertebrates (1–5). The molecular mechanisms that govern these events are complex and not fully described, but there is evidence that several of the TH-regulated effects are exerted through a genomic mechanism mediated by thyroid hormone receptors (TRs) (6), which are members of the nuclear receptor superfamily. In vertebrates, TRs are encoded by two distinct genes denominated thyroid hormone receptors alpha (*THRA*) and beta (*THRB*), which in turn are transcribed into several TR isoforms with tissue- and species-specific functions (7, 8). Aside from T3 (3,3′,5-triiodo-L-thyronine), T2 (3,5-diiodo-L-thyronine) also functions as an important TR ligand known to bind preferentially to different Tr isoforms, at least in teleosts (9), suggesting that each of these ligands can modulate different transcriptional processes.

In a comparative scenario, zebrafish have become an invaluable tool to start unraveling some of the mechanisms involved in vertebrate developmental processes. For example, during segmentation [10–20 h post fertilization (hpf)] an anteroposterior embryo is well-defined, and somites, tail, and a rudiment of the head and eyes can be observed (10). Some transcription factors that regulate cell fate and differentiation are regulated in a fine way before segmentation starts (5–10 hpf). During gastrulation (5–10 hpf), epiboly, internalization and germinal layer formation, as well as a correct positioning of dorso-ventral, antero-posterior, and left-right axis of embryo occur (11, 12), and several genes involved in cell fate and organogenesis start expression during this stage. Trs are known to function as the TH signal modulators during zebrafish development and in concert their coding mRNA is present in the fertilized egg in high concentrations during the first 6 hpf, after which *thr* mRNA decreases to low or non-detectable levels until 24 hpf (13, 14). The fact that *thr* mRNAs exhibit their highest levels concomitantly with those of intra-ovum THs (15) suggests that both Tr and ligand are from maternal origin and that these can be functional before embryonic transcription of *thr* and the appearance of the thyroid gland for embryonic TH synthesis. Tr manipulation results in stronger effects than those from exogenous TH administration. Indeed, overexpression of Trα has shown dramatic effects upon craniofacial development (16), and recently, human dominant-negative TRs were employed to determine the role of these isoforms during development (14, 17).

The objective of the present study was to further understand the role of THs and their receptors during early development. To that end, we evaluated the effects of exogenous T3 and T2 exposure during embryogenesis as well as disrupted *thr* expression using the CRISPR/Cas9 methodology. Also, we analyzed the expression of some early development genes and identified if they were TH-responsive and direct *thra*- or *thrb*-mediated TH targets.

## Materials and Methods

### Animals

Adult zebrafish (*Danio rerio*) were purchased from a commercial pet store and acclimatized to laboratory conditions: flow-through system with tap water at 28°C and a photoperiod 16:8 (light:dark). Embryos were obtained from natural mating, washed with tap water and cultured with E3 standard medium containing 5 mM NaCl, 0.17 mM KCl, 0.33 mM CaCl_2_, 0.33 mM MgSO_4_ and methylene blue. Un-injected embryos (UN) were immediately placed into an incubator at 28.5°C. All zebrafish were maintained and handled in accordance with protocols approved by the Ethics for Research Committee of the Instituto de Neurobiología at the Universidad Nacional Autónoma de México (UNAM).

### Preparation of sgRNA and mRNA Cas9

The zebrafish *thraa, thrab*, and *thrb* gene sequences were obtained from the zebrafish information network (www.zfin.org), and the different isoforms were verified on Ensembl (www.ensembl.org).

A well-established protocol was followed for genome editing (18). The main steps are briefly described: The scoring algorithm designed and tested in zebrafish CRISPRscan [www.crisprscan.org; (19)] was used to design sgRNAs. The selected target regions and selected guides for *thraa* and *thrab* are illustrated in Supplementary Figure 2 and Table 1. In the case of the *thrb* gene, *two* guides were used: sgRNA *l- thrb*+, which only targets the newly identified super long *thrb* (*l- thrb*+, see below), and sgRNA thrb^*^, which potentially targets three distinct isoforms (the well-described long (l-)Trβ1 and short (s-)Trβ1 isoforms and the newly described l-Trβ+) (Supplementary Figure 3). sgRNA was synthetized by *in vitro* transcription using T7 Quick High Yield RNA Synthesis Kit (New England Biolabs). Cas9 mRNA was also synthetized by *in vitro* transcription using an *Xba*I-linearized pT3TSn-Cas9n plasmid as template through mMESSAGE mMACHINE T3 kit (Life Technologies). sgRNAs and mRNA Cas9 were purified by ethanol precipitation and resuspended in RNAase free water. For the CRISPR/Cas9 microinjection, Cas9 mRNA and sgRNA mix was prepared and zebrafish embryos were injected directly with a final volume of 1 nL equivalent to 100 and 20 pg of Cas9 and sgRNA per embryo, respectively.

Table 1Primers and templates.

**Table:** 

**Gene target**	**Sequence identifiers**	**Forward primer (5′-3′)**	**Reverse primer (5′-3′)**	**Primer position**	**Length (bp)**
**REAL TIME PCR PRIMERS**					
*dio2*	NM_212789.4	GCAGCGCATGTTAACCACAG	GTTGTGGGTCTTACCGCTGA	Exon 1–2	160
*dio3a*	NM_001256003.1	CGCTCGTGTGTCTGCTCATT	CAGAGACTCCCAGCTGAACA	Exon 1	175
*thraa*	NM_131396.1	ATGGAAAACACAGAGCAGGAG	AGGAACAGAGATGCTCTTGTC	Exon 2–4	132
*thrab*	ENSDART00000153187.2	GGATGGAAATAAGGTGAATGGAAC	GGTAGTGATATCCGGTAGCTTTG	Exon 3–5	210
*s-thrb1*	NM_131340.1	AGAAGACTGTATGGGATCGAC	GTCTTCTGGCAGGAATTTGCG	Exon 9–10	134
*l-thrb1*	ENSDART00000151766.3	AGAAGACTGTATGGGATCGAC	GGCTTGGCTTCCTTCACCC	Exon 7–8	154
*mct8*	NM_001258230	GTTCGGGAAGATCGGAGACC	AACACGGCACACTGAGGAAT	Exon 21–22	111
*ets1*	NM_001017558.1	GAGATTTCTGGACCTGGCAC	GAAATATTCGGAGGGATAGCGG	Exon 4–5	145
*eve1*	NM_131114.1	GGGAACAGCTGACTCGTCTC	TGTCCTTCATTCTCCGGTTC	Exon 2–3	141
*fgf1b*	NM_001105278	CATGAGACTGGACTATACCTTGC	GTCCTGATATCTCTGCGAACG	Exon 2–3	138
*foxd3*	NM_131290	GTCCCGTCAAATATCATCTCCG	GCCTATAGTTCGTGCTGTATCG	Exon 2–3	150
*msx1a*	NM_131273.1	TCACACCCGTTTCACAGAC	CGGCAAACTTCACAAGTCAC	Exon 2–3	147
*sox10*	NM_131875.1	TCAATATCCGCACCTGCAC	CGCTTATCCGTCTCGTTCAG	Exon 2–3	82
*pax7a*	ENSDART00000172008.3	TCTGCAAAGTTCCTCCGGATT	CTGCAGTGCACAATGCCAAA	Exon 1	310
*zic1*	NM_130933.2	CTACACACATCCCAGTTCTCTC	TCTGGTTTTCTGTGGAAGGG	Exon 2–3	143
*mbpa*	ENSDART00000052556.8	GAGGAGACAAGAAGAGAAAGGG	GAAATGCACGACAGGGTTG	Exon 1–2	83
*mpz*	NM_194361.2	ACCTGTGATGCCAAGAACC	TTGCCACAACGAGGATCA	Exon 3–4	148
*olig2*	ENSDART00000060006.5	CGAGTGAACTGGAATAGCCTTAC	GCTCGTGTCAGAGTCCATG	Exon1–2	134
*plp*	ENSDART00000003514.8	ACACTGTTAACGTCCTGTCAG	CTGGTGCTTTGCATATGTTGG	Exon 4–5	147
*lsm12b*	NM_213148.1	AGTTGTCCCAAGCCTATGCAATCAG	CCACTCAGGAGGATAAAGACGAGTC	Exon 3–4	300

**Table:** 

**Gene target**	**Sequence identifiers**	**Forward primer (5′-3′)**	**Reverse primer (5′-3′)**
**gDNA PRIMERS**			
*thraa*	ENSDARG00000000151	TGTCAGATGGCCAAATGGAGT	CTGGTTGCGGGTGATTTTGT
*thrab*	ENSDARG00000052654	AGCTCTCGGAGCTGAAAGTG	ACCAGTGTAAGGAATAAAGTTGCT
*thrb (l-thrb+)*	ENSDARG00000021163	GACATAGCCCATGGTGTAAG	CTTTCTTATGTGGCCCTTGC
thrb (thrb^*^)	ENSDARG00000021163	GCATGGCTACAGACTGTAAG	GTTGTCAACAGGGAAGAGAC

**Table:** 

**Templates for** ***in vitro*** **transcription of sgRNAs**	
*thraa*	taatacgactcactataGGAGCGGTAATGATAGCCAGgttttagagctagaa
*thrab*	taatacgactcactataGGGAAAGAACAGCCAGTGTTgttttagagctagaa
*l-thrb+*	taatacgactcactataGGGTGAGTTATGCACCATGGgttttagagctagaa
thrb^*^	taatacgactcactataGGGAGAACCGTGAACGCCGAgttttagagctagaa
*Generic for template assembly*	AAAAGCACCGACTCGGTGCCACTTTTTCAAGTTGATAACGGACTAGCCTTATTTTaacttgctatttctagctctaaaac

### Microinjection

Embryos for microinjections were prepared according to Rosen et al. (20). One-cell stage zebrafish embryos were injected directly with a final volume of 1 nL of vehicle or working solutions using 1.5 OD/1.12 ID thinwall capillars (World Precision Instruments) and a Pneumatic PicoPump (PV 820; World Precision Instruments). To calculate intra-ovum TH concentration, an intra-embryonic volume of 170 nL was estimated. Groups of around 50 eggs were injected with the corresponding guide, either TH or vehicle, which consisted in DEPC water for CRISPR/Cas9 experiments and 10^−7^ N NaOH for TH treatments. Three independent experiments were performed per group.

### DNA Extraction

A HotSHOT modified protocol (21) was used to extract larval genomic DNA, in which 45 μL of 50 mM NaOH were added to each individual larva, followed by a 30 min incubation at 95°C. The samples were cooled at 4°C, and 5 μL of 1 M Tris-HCl, pH 8.0 were added to neutralize the solution. The samples were centrifuged to pellet debris and 5 μL of the supernatant were used for 50 μL PCR reactions.

### Crispant Verification

To verify that the zebrafish larvae contained the desired gene mutations, a fragment of *thraa, thrab*, and *thrb* genes that included the CRISPR/Cas9 target sites was amplified. To this end, a pair of primers for each gene was designed (see Table 1). An equal volume of DNA from 8 larvae of the same experimental condition were mixed (un-injected wild type and injected) for PCR amplification using Platinum Taq DNA Polymerase (Invitrogen) and subsequently column-purified (DNA Clean & Concentrator ™; Zymo Research). Purified PCR amplicons were either directly sequenced or used for subcloning in the TA vector pGEM-T Easy (Promega) to analyze the mutant allele populations from the injected larvae. Isolated colonies resulting from competent bacteria transformations were plasmid extracted and sent to sequencing using the universal primer T7.

### Quantitative PCR

The expression of selected genes was quantified in native, TH-treated and crispant larvae. To that end, total RNA was extracted from 0 un-injected zebrafish fertilized eggs and 9 hpf embryo pools (8-16) with Trizol Reagent (Life technologies). RNA was reverse transcribed with RevertAid Reverse Transcriptase (Thermo Scientific) from 1 μg of total RNA and 0.5 μg oligo (dT). Specific oligonucleotides were designed with Real-time PCR tool IDT. PCR products were obtained using a proofreading DNA polymerase for 10′ at 95°C, 10″ at 95°C, 10″ at 61°C and 10″ at 72°C for 40 cycles and were cloned into pJET1.2/blunt vector (Thermo Scientific). Constructs were verified by sequencing, and standard curves that ranged from 10^5^ to 10^9^ molecules/μL were prepared. In all cases, reactions contained 1 μL of reverse transcribed reaction, 6 μL Maxima SYBR Green/ROX qPCR Master Mix (Thermo Scientific) and 250 or 500 nM forward and reverse oligonucleotides in a final volume of 12 μL. A Step One instrument was used for detection and data analysis according to the manufacturer's instructions (Applied Biosystems). The absolute mRNA concentration was expressed as molecules per microgram of total mRNA used in RT reaction and obtained by interpolation with the standard curve and normalized with reference gene lsm12b (22) in each experimental sample. Oligonucleotides used for gene quantifications are listed in Table 1.

### Statistical Analysis

Results were analyzed using ANOVA coupled to a Tukey *post-hoc* test (control vs. treatments) and GraphPad Prism 7. Differences were considered statistically significant at *P* ≤ 0.05.

## Results

### Effects of Exogenous T3 and T2 Exposure During Embryogenesis

**Figure 1:**
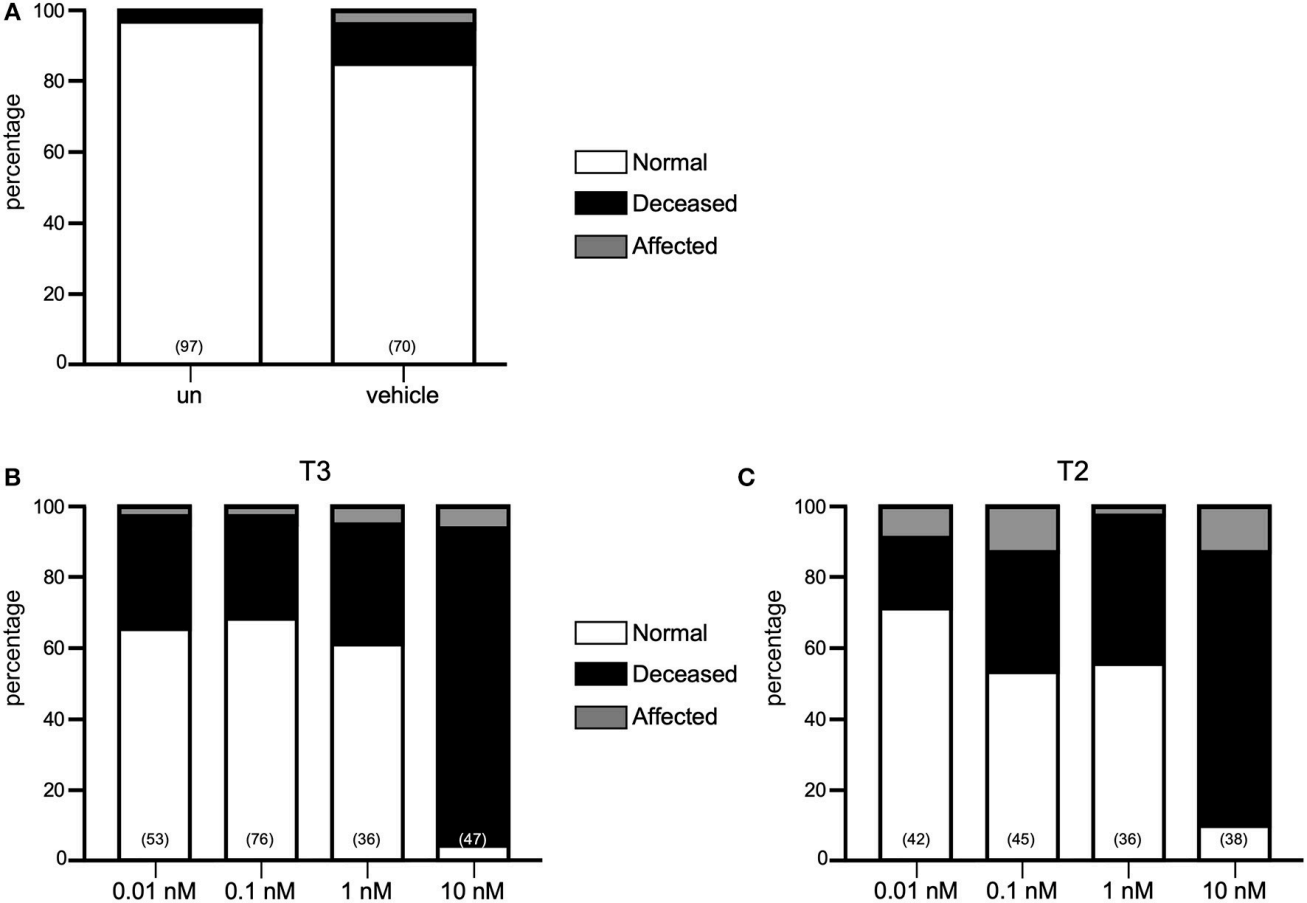
Effect of THs in zebrafish development. **(A)** Percentage of normal, affected and deceased larvae in un-injected (UN), vehicle or TH treatments. Neither T3 **(B)** nor T2 **(C)** had effects upon morphology at 0.1–10 nM but is toxic at 10 nM.

Until now, only immersion administration protocols have been used to deliver exogenous TH into the teleost embryo (23, 24). For this study we microinjected one-cell stage zebrafish embryos with T3 or T2 at concentrations ranging from 0.01, 0.1, 1, and 10 nM and observed for effects on the general body plan or on mortality during the first 48–50 hpf. As depicted in Figure 1, 19–41% and 30–33% of mortality was observed after the injection of 0.01, 0.1, and 1 nM of both T3 and T2, respectively, however, mortality increased to 76–89% when embryos were injected with 10 nM of either hormone, clearly showing toxic effects. In all cases, the percentage of affected embryos was similar to those vehicle-injected (below 6% for T3 and 13% for T2), suggesting that the microinjection did not influence development, as well as that the early exposure to the hormone alone was not sufficient to elicit effects at least upon the phenotype of the embryo (Figure 1). Since transactivating assays showed that zebrafish TRs were activated with 0.1 nM (Supplementary Figure 1), and a slightly lower mortality rate was observed with this concentration compared to 1 and 10 nM, subsequent experiments were performed with 0.1 nM of either T3 or T2.

#### thraa but Not thrab Impacts Early Zebrafish Development

**Figure 2:**
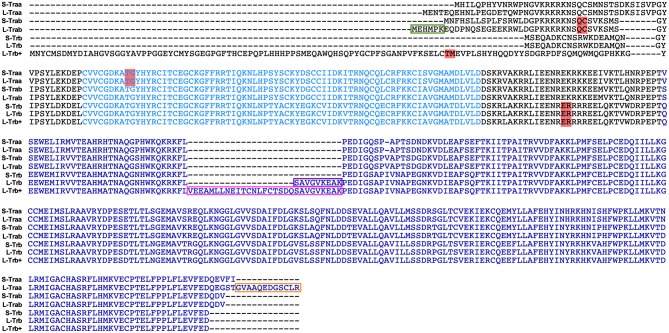
Primary sequence of thyroid hormone receptor isoforms from zebrafish analyzed in this study. The alignment depicts the DBD (light blue) and LBD (Blue)which are highly conserved. Isoforms were denoted as short (S) or long (L), according to the respective counterpart isoform. The teleost-specific insert of 9 amino acids from l-Trβ1 and the newly identified 9 plus 20 amino acid sequence from l-Trβ+ are denoted in purple and pink, respectively. We highlight in red the sgRNA targets for each isoform. The alignments were prepared using MEGA 8.

**Figure 3:**
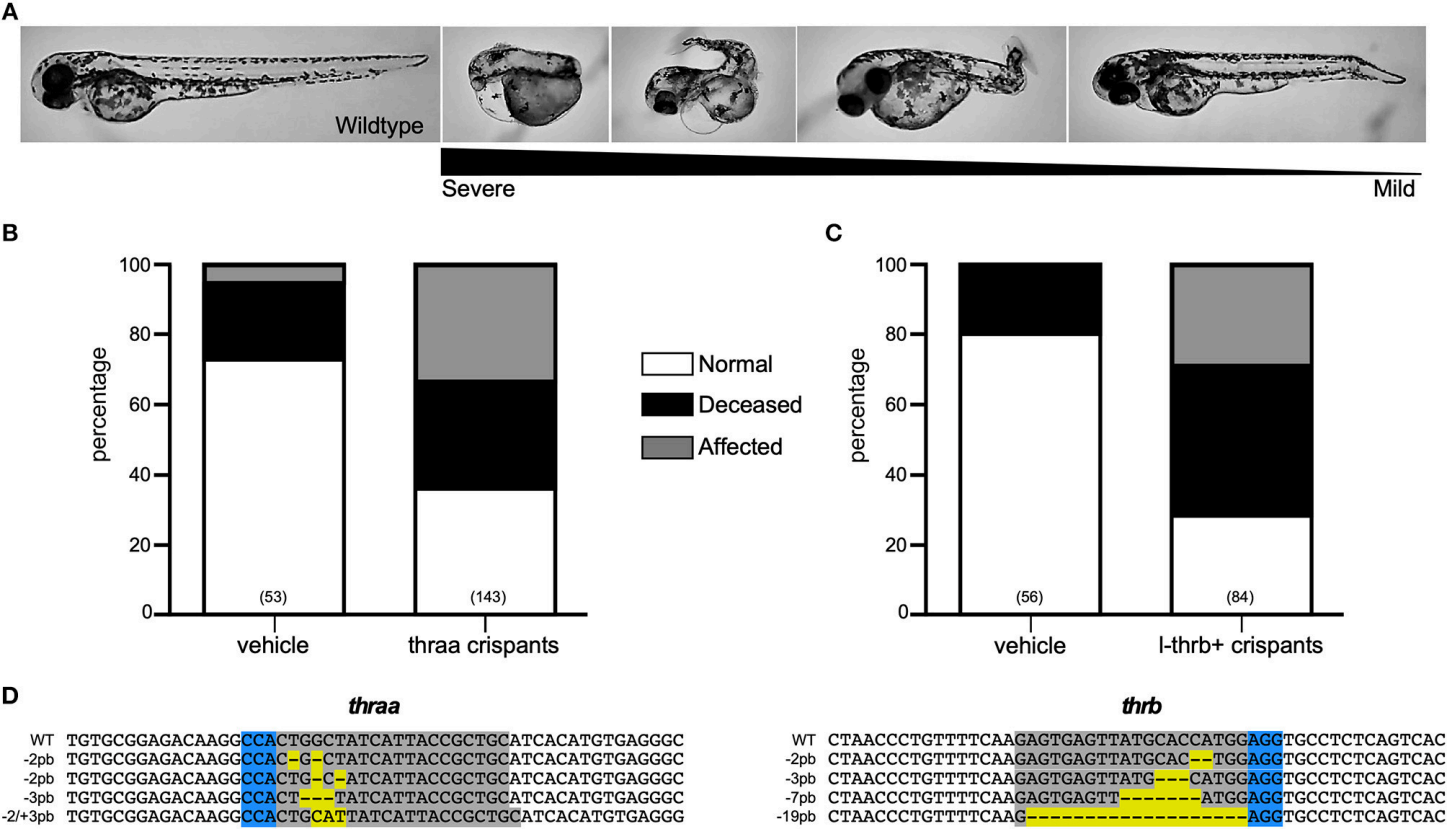
Characterization of *thraa* and *l-thrb*+ crispants. **(A)** *thraa* and *l-thrb*+ crispants show diverse phenotypes with several degrees of body plan affectations as compared with wild-type at 3 dpf. Various phenotypes were observed due the mosaicism of the mutation. **(B**,**C)** Represent the percentage of normal, affected and deceased larvae after sgRNA and Cas9 treatment. **(D)** DNA sequence of sgRNA target site for CRISPR/Cas9 is denoted in gray, whereas PAM sequence is denoted in blue. Insertion-deletions (indels) are highlighted in yellow.

**Figure 4:**
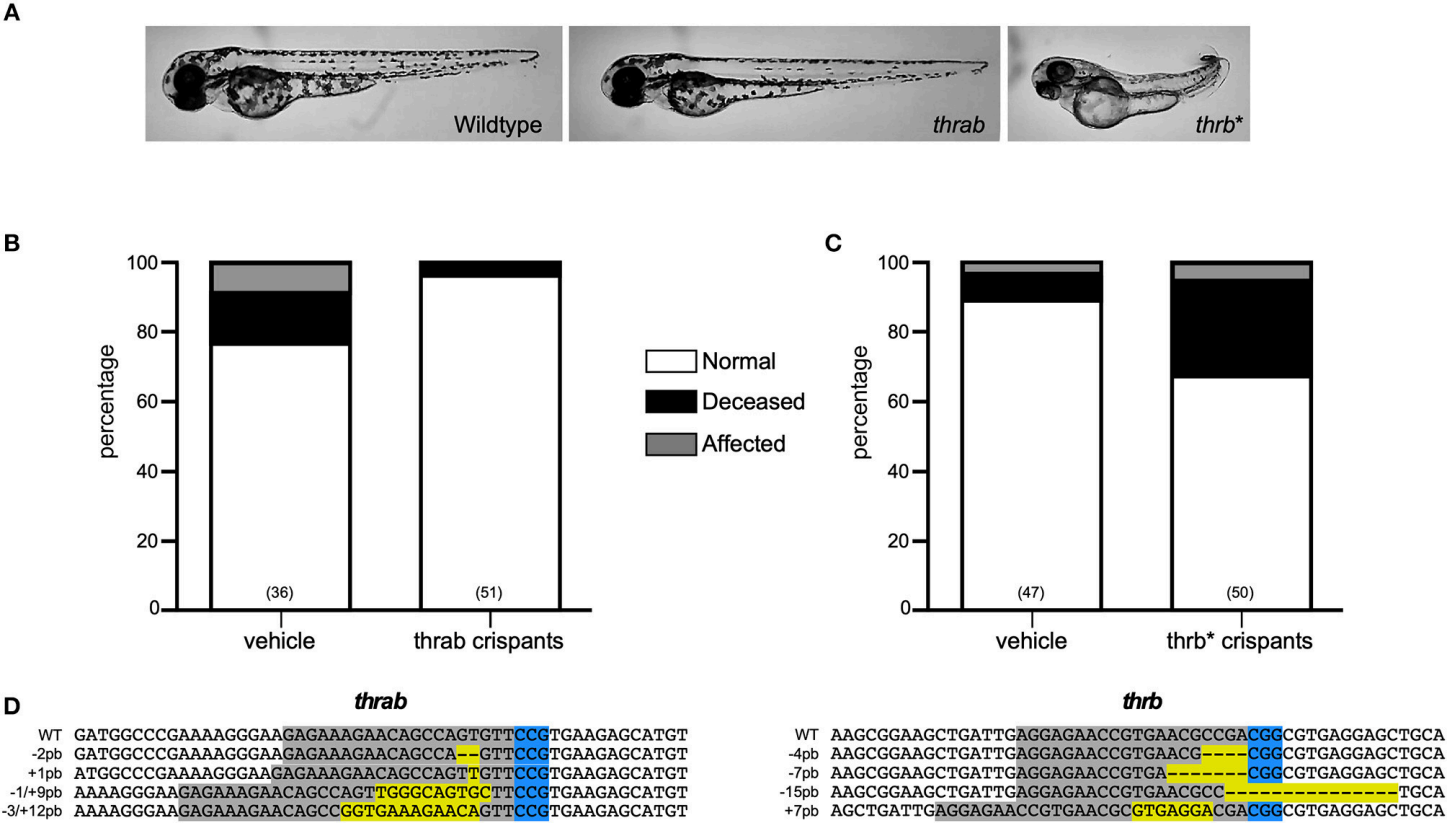
Characterization of *thrab* and l-thrb^*^ crispants. **(A)** *thrab* crispants do not show effects upon zebrafish morphology, whereas l-thrb^*^ crispants generated with an sgRNA able to target all *thrb* isoforms showed a low percentage of affected phenotypes characterized by laterality. **(B**,**C)** Represent the percentage of normal, affected and deceased larvae after sgRNA and Cas9 treatment. **(D)** The DNA sequence of sgRNA target site for CRISPR/Cas9 is denoted in gray, whereas PAM sequence is denoted in blue. Insertion-deletions (indels) are highlighted in yellow.

Two *thra* genes have been described in teleosts, but from these genes, only *thraa* has been more extensively studied. In zebrafish, two *thraa* transcripts have been described, which differ in the presence of an extension of around 12 amino acids in the C-terminal domain (Figure 2). In contrast, only one transcript has been described for *thrab*, however, we identified two transcripts in Ensembl that differ by 6 amino acids at the N-terminal of the protein (Figure 2). As mentioned above, for this study, guides to disrupt *thra* genes were designed to target the two identified isoforms per gene. Designed sgRNA guides were effective to produce a variety of mutations reflected in the electropherograms from crispant DNA with respect to that from wildtype embryos (Supplementary Figures 2, 3). Our results show that approximately 35% of the *thraa* crispant larvae presented a clear loss of symmetry and laterality (asymmetric size and position of external morphology mainly of eyes, head, and tail) observed as early as 24 hpf when the effects were severe, or from 3 to 4 days post-fertilization in mildly to moderately affected larvae when the body plan had taken form (Figure 3A; Supplementary Figure 4). Moreover, this group of crispants exhibited a 30% mortality (Figure 3B). In contrast, when *thrab* crispants were analyzed, no effect was observed in larvae (Figures 4A,B), and mortality was only 5.5%. Altogether, these results strongly suggest a novel role for *thraa* in the body plan development program, whereas *thrab*, although expressed in early development (13, 25), does not seem to participate in these processes.

#### Zebrafish L-trb+ Crispants Show Impaired Embryogenesis

As mentioned, several functional teleost-specific *thrb* isoforms have been identified. Of these, the most representative are two gene products that contain or not an insertion of nine amino acids in the ligand binding domain (LBD) of the protein and that have been referred to as long or short thyroid hormone receptor β1 (S- or L-Trβ1). However, some metamorphic species have been shown to express an additional *thrb* isoform that contains 20 amino acids adjacent to the 9-amino acid insert, and that has been denominated L-Trβ1+ (8). Interestingly, we identified (Ensembl) an isoform that had not been previously identified in zebrafish that contains a 111 amino acid N-terminal fragment, similar in length to that of the human Trβ2, but with only 26% of conserved amino acids within this region (Figure 2). The low sequence identity in the fragment raises the doubt that the isoform could indeed be a Trβ2. Furthermore, this Trβ isoform contains the 29-amino acid insert, as described for the metamorphic species (L-Trβ1+). Given the ambiguity to clearly identify this isoform in terms of sequence identity, in this study we have denominated it L-Trβ+.

To unravel the putative role of this as well as the other two *tr*β*1* during early development, crispants for the three isoforms were generated by using the guide thrb^*^, as well as crispants that were directly targeted to disrupt *l-trb*+. Surprisingly, only crispants specifically generated to disrupt *l-trb*+ presented effects in 30% of larvae, showing a loss of symmetry and laterality in the same manner as *thraa* crispants (Figures 3A,C). In contrast, crispants resulting from a target site that disrupts a shared *Tr*β*1* sequence (Figure 2) only showed minor effects as when using the guide that disrupts *l-trb*+ alone (Figures 4A,C). Although intriguing, these results evidence a clear functional role of the novel *l-trb*+ isoform at least during early development. No morphological defects were observed in 60% of crispants generated with sgRNA thrb^*^, an observation that could result from the nature of the sgRNA target site, which renders transcripts that still contain the DNA-binding domain (DBD), possibly allowing the truncated protein to bind to TH-responsive elements in target gene promoters and repress gene transcription. However, the 32% mortality observed in this group could also correspond to the population where *l-trb*+ expression was most affected, further suggesting that the canonical *thrb* isoforms do not appear to be involved in early developmental events.

#### Changes in Development-Related Genes at 9 hpf

Both *thraa* and *l-thrb*+ crispants showed a clear loss of symmetry and laterality, visualized at 24 hpf when a well-defined antero-posterior pattern was observed in control larvae. As previously mentioned, critical events that determine cell fate for these developmental stages, like epiboly, internalization and germinal layer formation, start in the gastrula (approximately 5–10 hpf), (10, 26). Thus, we hypothesized that some genes that are expressed during early development could indeed be TH targets, with Tr-specific signaling pathways. To prove our hypothesis, we chose 9 hpf embryos, which were at the onset of segmentation, to analyze the expression of sets of genes known to be part of TH signaling (*dio2, dio3, mct8, thraa, thrab, s-thrb1, l-thrb1*), and genes involved in symmetry and laterality (*eve, fgf, zic, pax 7, msx, foxd3, sox 10, ets*) and in myelination (*mpz, mbp, olig 2, plp1b*) (Table 1). Since myelination does not start until 48 hpf in zebrafish (27), the latter set of genes was included as a negative control group. This screen was performed in fertilized un-injected eggs to determine gene expression at time zero and in the 9 hpf vehicle-injected embryos or embryos treated with T3 and T2, as well as in crispants generated for all *thr* isoforms. As 9 hpf is too early to detect body plan malformations, the mRNA pool samples of crispants were heterogeneous since we were unable to distinguish between affected and normal embryos. Nonetheless, we were still able to detect clear changes in mRNA expression in the different experimental groups, with clear statistical significance compared to controls. Exogenous TH exposure influenced the regulation of several genes at this stage of development, showing that the expression of TH-responsive genes is receptive to TH regulation during gastrulation. The fact that T2 had an effect upon gene regulation suggests that in this developmental stage, as in the juvenile and adult stages, T2 is a relevant TR alternative ligand (28, 29) (Lazcano et al. under review)^1^.

**Figure 5:**
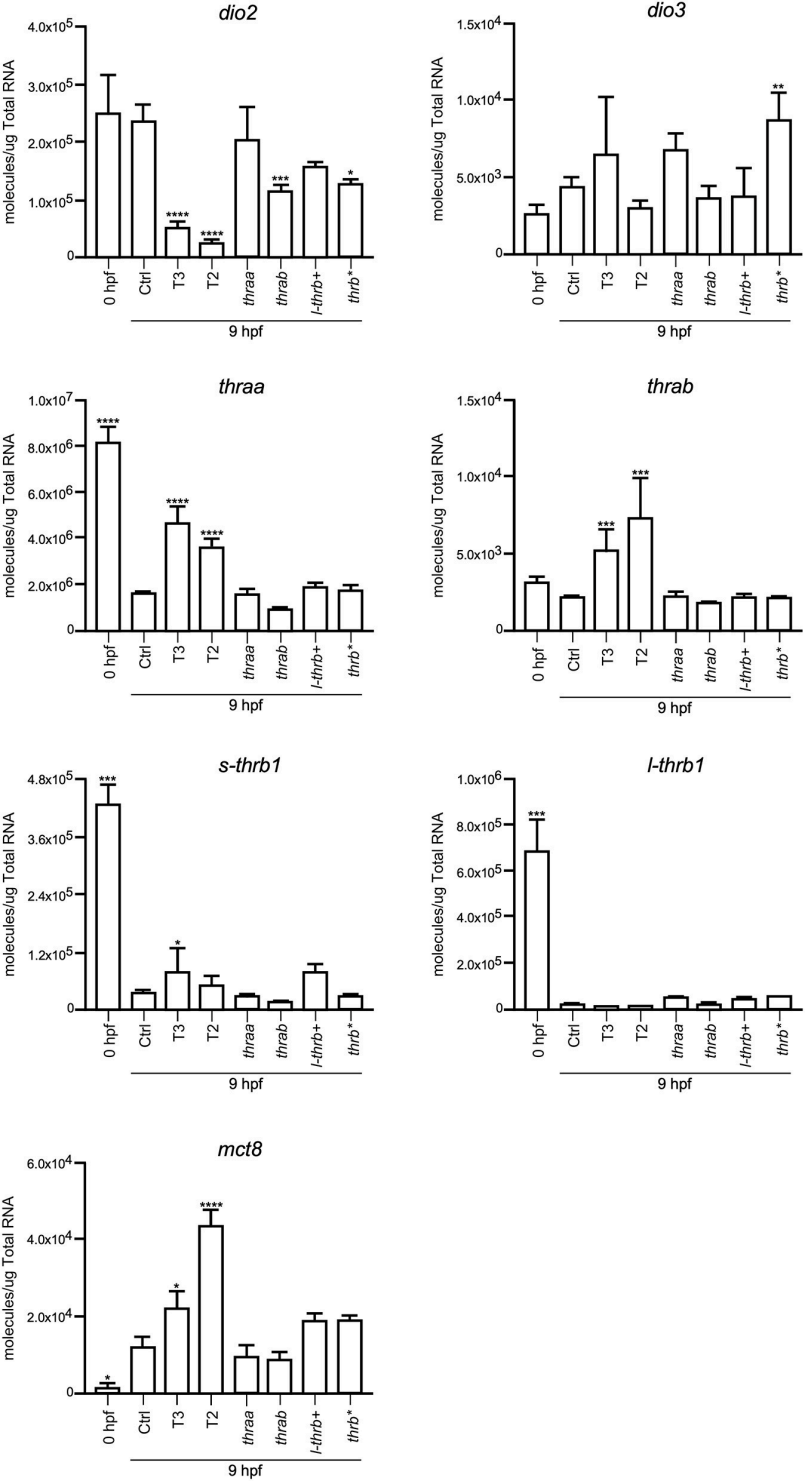
Embryonic mRNA expression of TH-signaling genes *dio2, dio3, thra, l-thrb1, s-thrb1, and mct8*. Total RNA was extracted from 9 hpf zebrafish embryo pools (8–16) for qPCR. The plots show mean values ± SEM results of three independent biological experiments. Statistical analysis was performed using one-way ANOVA coupled with a Tukey's multiple comparisons test with respect to control groups. Significance is indicated ^*^p < 0.05, ^**^p < 0.01, ^***^p < 0.005, ^****^p < 0.001.

**Figure 6:**
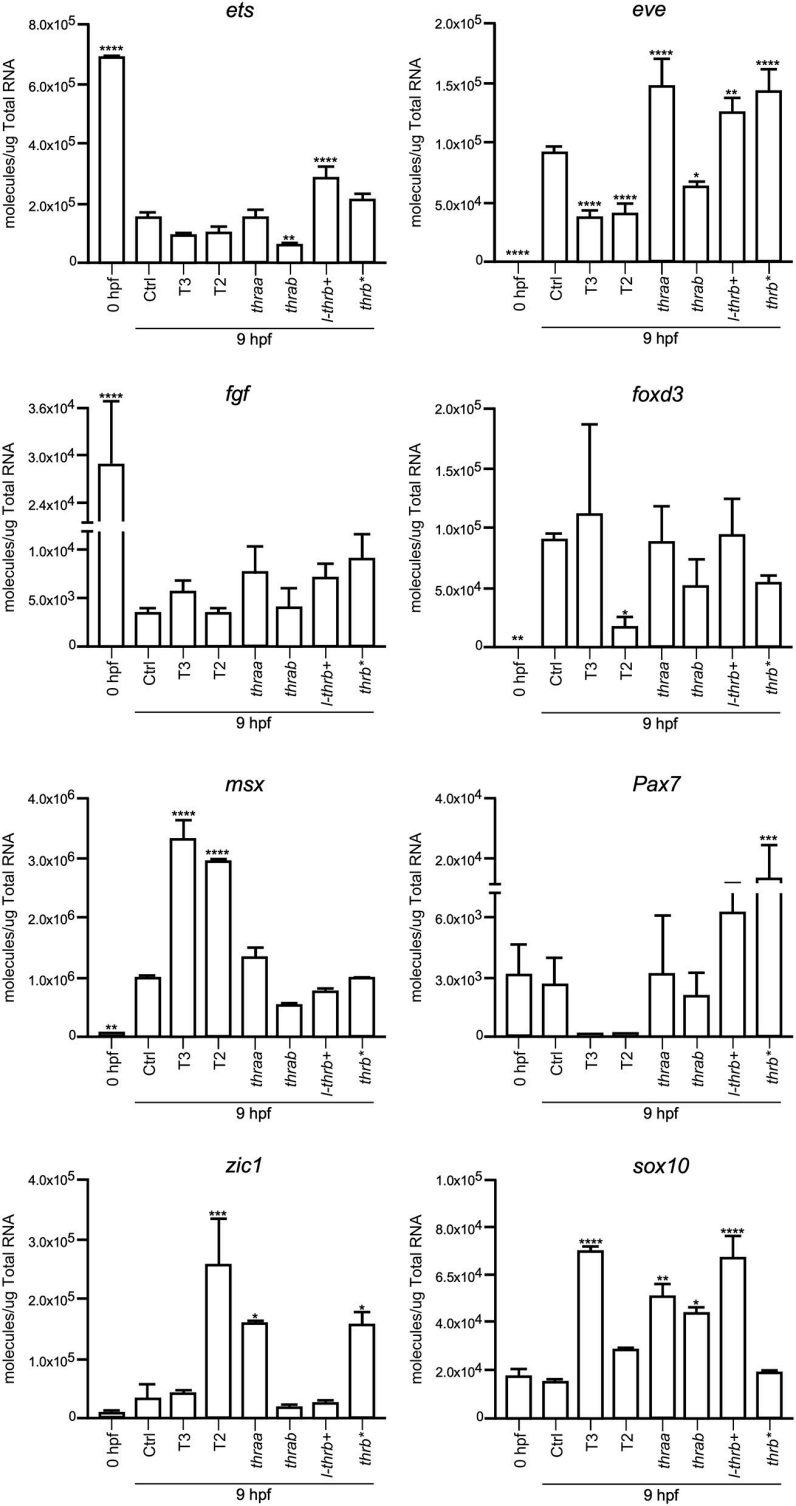
Embryonic mRNA expression of early development genes *eve, fgf, zic, pax7, msx, foxd3, sox10, and ets*. Total RNA was extracted from 9 hpf zebrafish embryo pools (8–16) for qPCR. The plots show mean values ± SEM results of three independent biological experiments. Statistical analysis was performed using one-way ANOVA coupled with a Tukey's multiple comparisons test with respect to control groups. Significance is indicated ^*^p < 0.05, ^**^p < 0.01, ^***^p < 0.005, ^****^p < 0.001.

**Figure 7:**
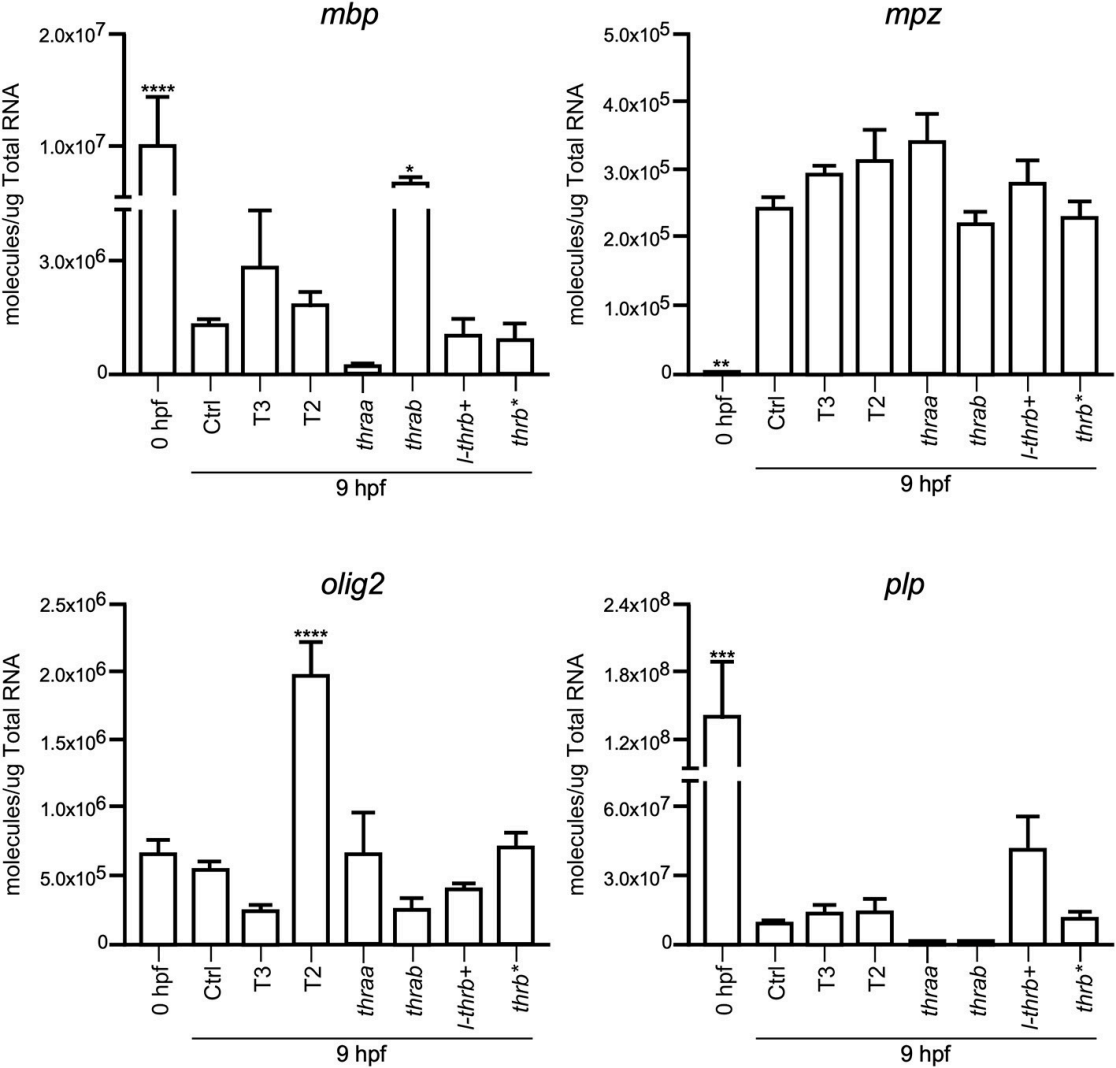
Embryonic mRNA expression of genes involved in myelination (*mpz, mbp, olig2, plp1b*). Total RNA was extracted from 9 hpf zebrafish embryo pools (8–16) for qPCR. The plots show mean values ± SEM results of three independent biological experiments. Statistical analysis was performed using one-way ANOVA coupled with a Tukey's multiple comparisons test with respect to control groups. Significance is indicated ^*^p < 0.05, ^**^p < 0.01, ^***^p < 0.005, ^****^p < 0.001.

As observed in Figures 5–7, mRNA from *dio2, thraa, s-thrb1, l-thrb1, mbp, ets*, and *fgf* was highly expressed at the stage of one-cell embryo (0 hpf), while mRNA expression of *mct8, mpz, eve, foxd3, msx*, and *zic* was not detected, showing the maternal origin of some transcripts. Indeed, transcripts from maternal origin are present in the oocyte, and zygotic transcription starts around 2 to 3 hpf. Other transcripts (*mct8, eve, foxd3, msx*, and *mpz*) were only detected after 9 hpf, evidencing onset of zygotic gene transcription (10). Furthermore, we identified genes that were up- (*thraa, thrab, msx*) or down-regulated (*dio2, eve, pax 7*) by both T3 and T2 at 9 hpf, as compared with vehicle-injected embryos, and genes that were specifically up-regulated by T3 (*sox10*) or by T2 (*mct8, olig2, zic*), as well as genes down-regulated by T2 (*foxd3*). We also identified genes that did not respond to TH treatment (*dio3, mbp, mpz, plp, ets, fgf, pax7*). Thus, out of the three sets of genes, at least those related to TH signaling and early development were indeed TH-responsive. The early exposure of T2 resulted in an up-regulation of *olig2*, a gene associated to myelination, suggesting a direct regulatory effect of this hormone. As an attempt to identify if the TH response was mediated by a specific *thr*, we analyzed the expression of the three sets of genes in *thr* crispants. Only *sox10, eve* and *zic* specifically increased their expression in *thraa* crispants, *sox10, ets* and *eve* in *l-thrb*+ crispants and *mbp* in *thrab*, suggesting that for these genes, *thraa, l-thrb*+, and *thrab* respectively, function as repressors of transcription. thrb^*^ crispants, where all *thrb* isoforms are targeted, exhibited an increased expression of *dio3, eve, pax7*, and *zic*, suggesting that the regulation of *eve* is mediated by *l-thrb*+ and that of the other genes by a different *thrb* isoform. In contrast, we observed a more diverse response on gene expression for *thrab* crispants: the expression of *mbp* and *sox10* was discretely increased, while that of *dio2, ets*, and *eve* decreased, suggesting that *thrab* could have other roles in later stages of development.

## Discussion

This is the first study in which *thr* crispants were analyzed in order to further understand the role of Trs in early development. We found that larvae from *thraa* and *l-thrb*+ crispants presented profound deleterious effects upon symmetry and laterality, suggesting early novel roles of these Tr isoforms in the body plan developmental program. We also explored the expression of early development genes known to be involved in symmetry and laterality in *thr* crispants and identified direct *thra*- or *thrb*-mediated TH targets.

Early TR expression has been described before gastrula in teleosts and birds (14, 30) and during the first trimester of gestation in the human fetal brain (31). As in the present study, *THRA* is the most representative TR expressed gene (14, 31), suggesting that TRα-mediated TH signaling regulates early developmental events in all vertebrates. It is thought that THs do not participate in early vertebrate development, only after neural tube closure (32, 33), however, and irrespective of the vertebrate species, the embryo is always exposed to low TH levels from maternal origin, and TR mRNA is present from the onset of development (13, 14). Studies aimed to analyze early ontogenetic TH effects in zebrafish have shown that these hormones accelerate hatching and pigmentation (23). In the present work we did not detect any of these effects with our administration protocol (data not shown), but we did observe changes in the transcription of several genes associated with TH signaling and to body symmetry. Indeed, *dio2, thraa, s-thrb1*, and *l-thrb1* were found to be highly expressed in one-cell embryos, emphasizing mRNA of maternal origin and the concomitant early onset of TH signaling. Furthermore, the expression of genes associated with body symmetry like *eve, foxd3, msx*, and *zic1*, was undetectable at the one-cell stage, but detectable in 9 hpf embryos evidencing the time-specific windows of gene expression during development. The fact that TH regulated some of these genes as early as 9 hpf clearly suggests that maternal THs regulate the transcription of genes involved in their own transport, metabolism and signaling, as well as the onset of cellular and molecular mechanisms involved in body symmetry and laterality. Given these observations, it was puzzling that no clear effects upon larval phenotype were evident after TH treatment. One possible explanation is that the fine down-regulatory effect that THs exert upon *dio2* expression (Figure 5) could serve as a protective mechanism from TH excess. Other possible explanations could involve particular TR action mechanisms yet to be elucidated.

The study of TRs and their ligands during development has focused on the central nervous system, where it is known that they are required to perform certain actions mainly related to neurogenesis and myelination (33–36) and other possible roles may have been overlooked. The zebrafish model has recently been used to address some relevant aspects of TR-TH function during development. Since teleosts underwent a specific round of genomic duplication, their genome contains several copies of genes that are absent in other taxa (37). In the case of zebrafish *thr*, the presence of a second copy of the *thraa* gene has been demonstrated and it has been called *thrab* (13, 25). The alignment of the primary sequence of the isoforms resulting from both *thra* genes shows differences only at the N- and C-terminal of the protein (Figure 2), while the DBD and the LBD show a high degree of identity, suggesting that all isoforms generated from the *thraa* and *thrab* genes could bind to DNA and ligate THs. In this study, the sgRNA designed to disrupt each gene modifies the open reading frame at the beginning of transcription, prior to the DBD, in both cases affecting the synthesis of all the possible isoforms for *thraa* and *thrab* that we identified by Ensembl. The resultant crispants carry the induced mutation, but the grade of penetrance is undefined because of the nature of the changes in the nucleotide sequence (monoallelic, biallelic in- or out of frame, in different cell types or occur over different time frames). Due to this crispant nature, and as determined by sequencing, we identified a great variety of mutated alleles in all four target genes analyzed (Figures 3D, 4D), most of them resulting in premature stop codons and frameshifts. This variety of alleles is reflected in the obtained phenotypic diversity (Supplementary Figure 4), however, only *thraa* crispants presented severely affected larvae. Indeed, *thraa* crispants showed high mortality, clear malformations, asymmetry and altered laterality, while *thrab* crispants did not. It is possible that *thrab* mutations could affect development in a different way, without evident abnormalities in body plan, whereas *thraa* could regulate the transcription of body plan genes during early development (see below). However, studies to corroborate the biological activity of the receptors encoded by *thrab*, as well as some possible roles during other stages of the zebrafish life cycle must be tested experimentally.

Although only a single copy of the *thrb* gene has been identified with the exception of eels (38, 39) and *Xenopus laevis* (40) which contain two copies of *thrb*, this gene has other characteristics that could confer biological plasticity, like the presence of several isoforms that differ in the N-terminal and/or the LBD (Figure 2). Zebrafish, for example, expresses the canonical Trβ1, homologous to the TRβ1 of mammals and other vertebrates, and also an isoform with a 9 amino acid insert in the LBD that has been previously characterized and is able to bind T3 as well as the alternative TR ligand T2 (9). More surprisingly, we also detected a zebrafish isoform that has an up-stream putative alternative start site of transcription different from the other *thrb* isoforms that results in a Tr with an extended N-terminal fragment similar in length but not in sequence identity to that of the human Trβ2, which additionally contains the 9 amino acid insert plus 20 amino acids more located adjacently, probably generated by alternative splicing (Figure 2). This isoform, which we denominated *l-trb*+, is the largest *thrb* identified thus far in teleosts and not previously characterized in zebrafish. As in the case of *thraa*, the disruption of *l-thrb*+ rendered larvae with deleterious effects upon symmetry and laterality that had not been previously described. In fact, the phenotype of both *thraa* and *l-thrb*+ crispants was so strong that no detailed scrutiny was needed to identify affected larvae. Thus, one of the most interesting findings of the present study was the isoform-specific effects that *thr* exerted during development.

In concert with the phenotypic assessments, when gene expression was analyzed in crispant embryos, we identified early development genes, like *sox10* and *eve*, that were specifically over-expressed in *thraa* and *l-thrb*+ crispants, suggesting that for these genes, these specific *thr* isoforms function as transcription repressors. This would be in agreement with the prevailing concept that during early vertebrate development, TR act mainly as dominant negatives when unliganded, at least for TH positively regulated genes (30, 31). It is noteworthy that *sox10* and *eve* are determinant for neural crest migration and tail extension (41, 42). Thus, precocious expression of *sox10* and *eve* could explain, at least in part, the dramatic malformations observed in *thraa* and *l-thrb*+ crispants. Interestingly, these two genes are regulated by THs in an opposite manner: T3 up-regulates *sox10*, while both, T2 and T3 down-regulate *eve*, showing the dynamic interplay between *thr* and ligands during early gastrulation. It would be very interesting for future experiments to isolate the different obtained alleles and look for differences or redundancy in its phenotype. Other genes that could be involved in these early developmental processes are *zic* and *ets*, which appear to be *thraa*- and *l-thrb*+-mediated, respectively. *zic* is involved in brain and somite development (43), while *ets* is a gene involved in vascularization (44). In contrast, with the exception of *mbp*, the expression of genes involved in the myelination process is not affected in the different crispants. The fact that *mbp* is up-regulated in *thrab* crispants suggest a not myelination-related function of this gene in early development. As previously mentioned, these observations are in concert with the notion that myelination starts around 48 hpf in zebrafish (27). The expression of other analyzed genes known to participate in zebrafish early development was not significantly modified. It is possible that these genes are not TH targets or that they act at different stages of development.

Overall, present results show that TH signaling participates in early zebrafish development. An interesting contribution of this study however is the identification of Tr isoform-specific mediated regulation of early gene expression. Thus, and although at this point, we cannot clearly decipher the respective contribution of each receptor isoform during early development, we did identify at least two genes whose regulation is specifically mediated by Trα and L-Trβ+, showing that the experimental strategies used in the present study will be useful to elucidate TR-specific functions.

## Data Availability

All datasets generated for this study are included in the manuscript and/or the supplementary files.

## Author Contributions

IL and RR-O performed the CRISPR-Cas9 experiments. PV performed the qPCR experiments. AO and IL directly participated in the planning and execution of this study and drafted the manuscript. All authors provided critical comments to the manuscript and revised the text. All authors of this research paper have read and approved the final version submitted.

### Conflict of Interest Statement

The authors declare that the research was conducted in the absence of any commercial or financial relationships that could be construed as a potential conflict of interest. The handling editor declared a past co-authorship with one of the authors AO.

## Footnotes

^1^Lazcano I, Hernández-Puga AG, Orozco A. Alternative ligands for thyroid hormone receptors. A molecular perspective. *Mol Cell Endocrinol*.
