# Supplementary figures and images for: Knock-Down of Specific Thyroid Hormone Receptor Isoforms Impairs Body Plan Development in Zebrafish
Source: Front Endocrinol (Lausanne). 2019 Mar 14;10:156. doi: 10.3389/fendo.2019.00156 (PMC6427925; doi:10.3389/fendo.2019.00156)

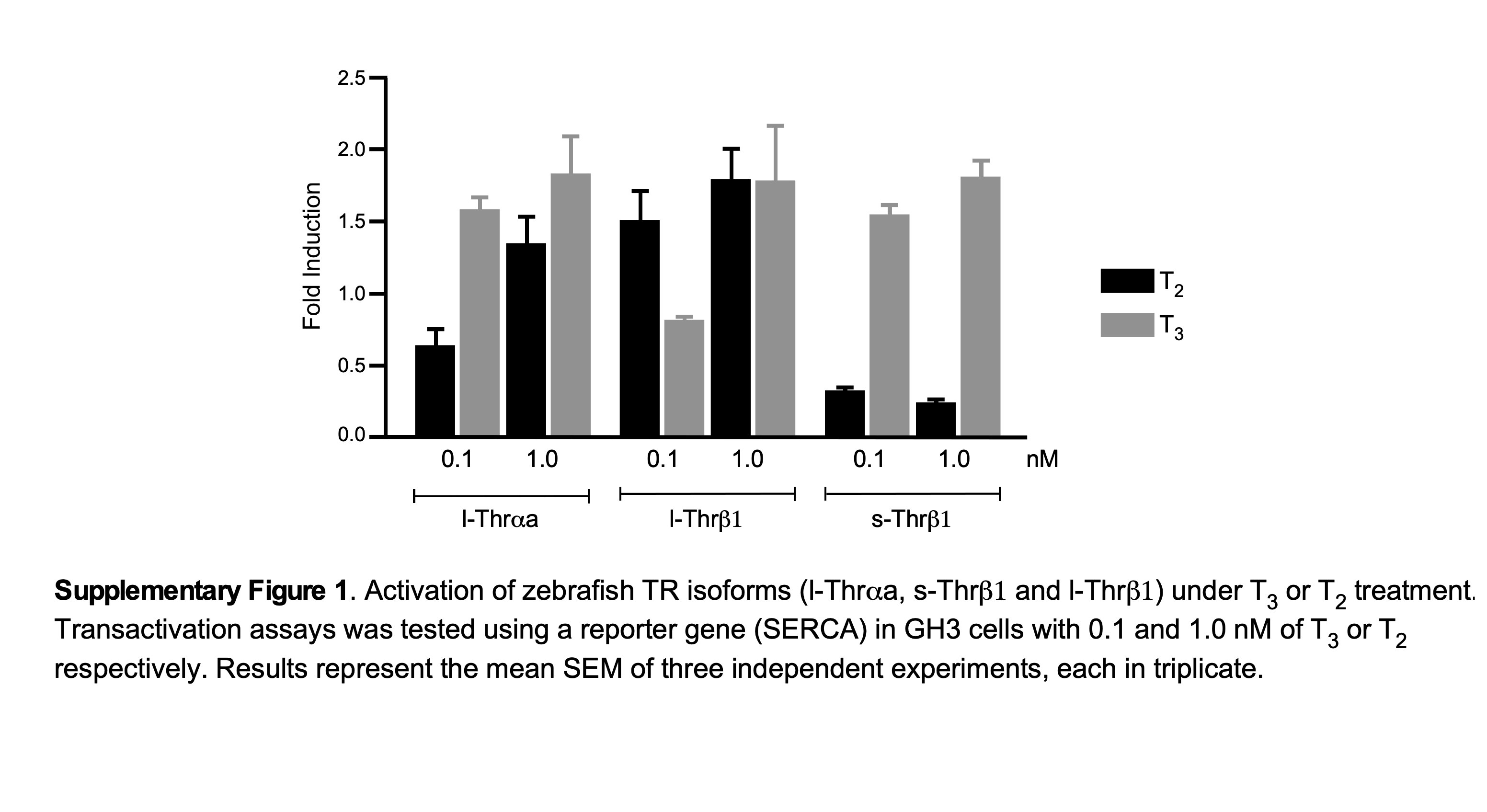

Supplement: Supplementary file 1 [file Image_1.TIF]

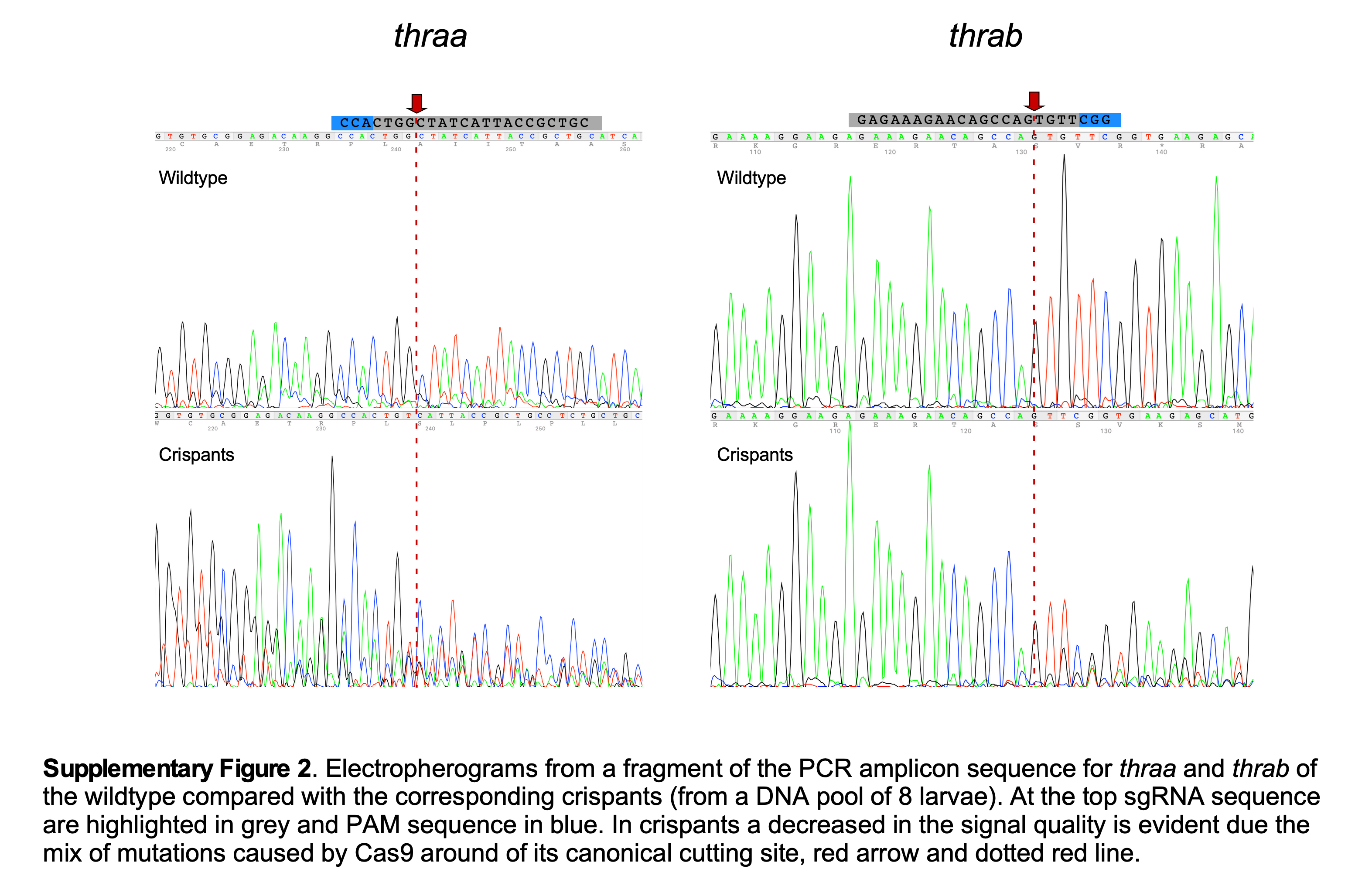

Supplement: Supplementary file 2 [file Image_2.TIF]

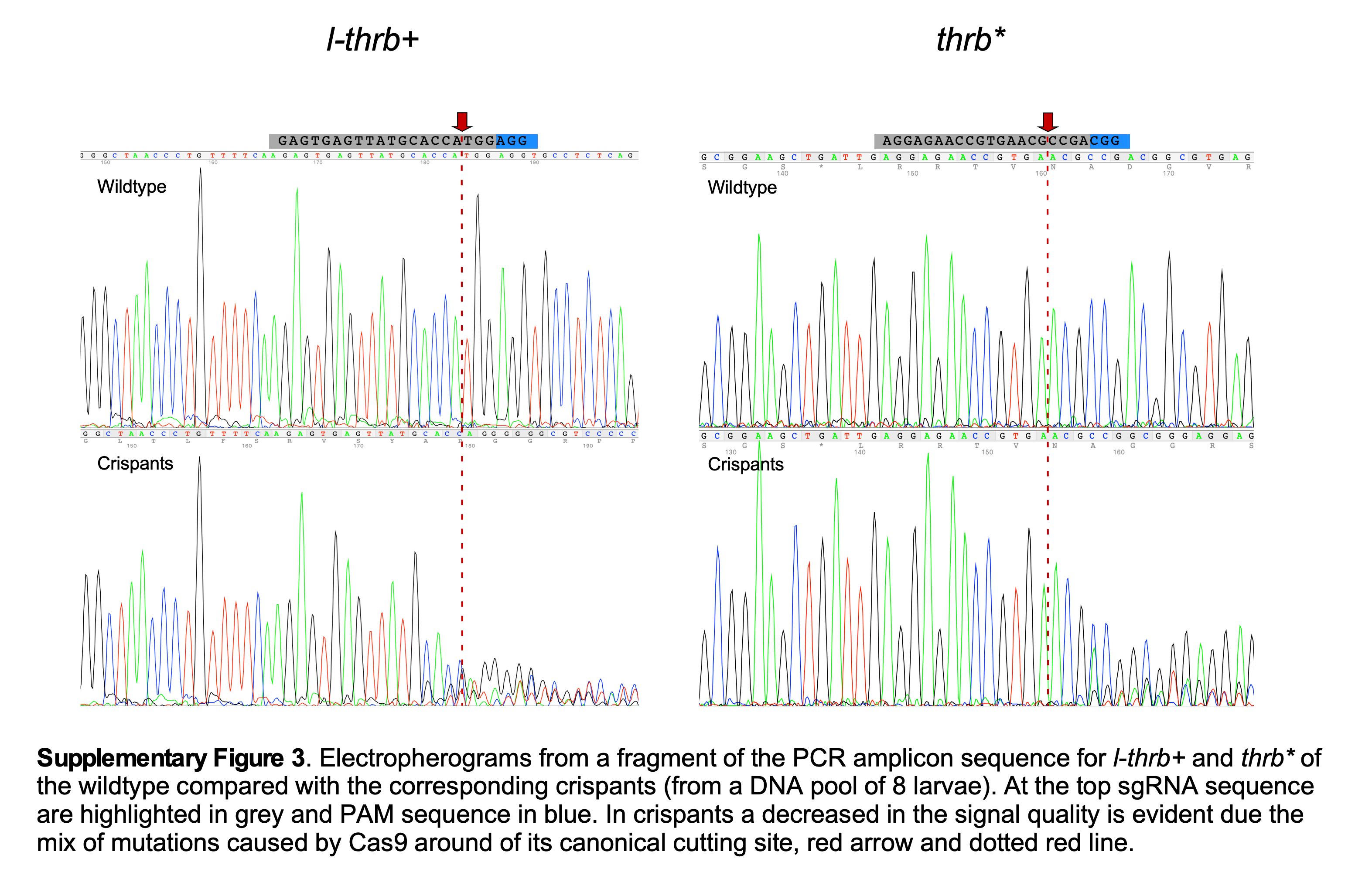

Supplement: Supplementary file 3 [file Image_3.TIF]

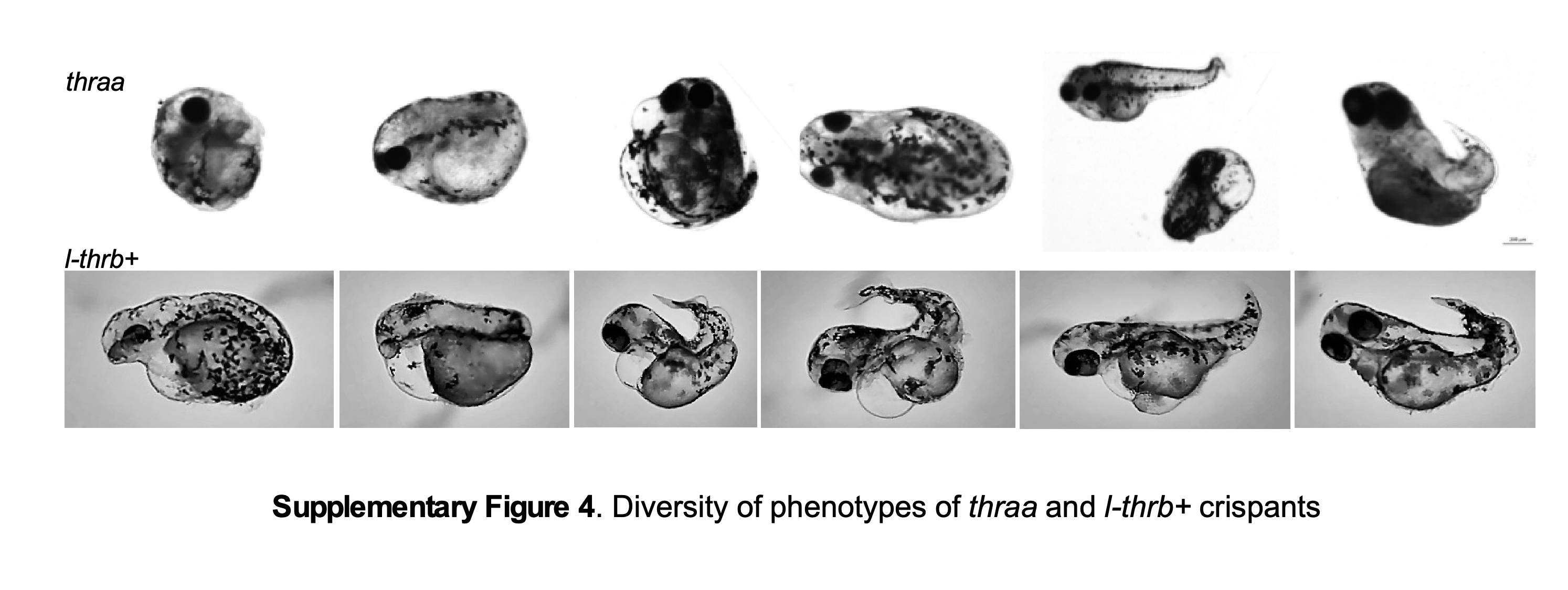

Supplement: Supplementary file 4 [file Image_4.TIF]
